# Sarcolipin deletion exacerbates soleus muscle atrophy and weakness in phospholamban overexpressing mice

**DOI:** 10.1371/journal.pone.0173708

**Published:** 2017-03-09

**Authors:** Val A. Fajardo, Daniel Gamu, Andrew Mitchell, Darin Bloemberg, Eric Bombardier, Paige J. Chambers, Catherine Bellissimo, Joe Quadrilatero, A. Russell Tupling

**Affiliations:** Department of Kinesiology, University of Waterloo, Waterloo, Ontario, Canada, N2L 3G1; University of Minnesota Medical Center, UNITED STATES

## Abstract

Sarcolipin (SLN) and phospholamban (PLN) are two small proteins that regulate the sarco(endo)plasmic reticulum Ca^2+^-ATPase pumps. In a recent study, we discovered that *Pln* overexpression (*Pln*^OE^) in slow-twitch type I skeletal muscle fibers drastically impaired SERCA function and caused a centronuclear myopathy-like phenotype, severe muscle atrophy and weakness, and an 8 to 9-fold upregulation of SLN protein in the soleus muscles. Here, we sought to determine the physiological role of SLN upregulation, and based on its role as a SERCA inhibitor, we hypothesized that it would represent a maladaptive response that contributes to the SERCA dysfunction and the overall myopathy observed in the *Pln*^OE^ mice. To this end, we crossed *Sln*-null (*Sln*^KO^) mice with *Pln*^OE^ mice to generate a *Pln*^OE^/*Sln*^KO^ mouse colony and assessed SERCA function, CNM pathology, *in vitro* contractility, muscle mass, calcineurin signaling, daily activity and food intake, and proteolytic enzyme activity. Our results indicate that genetic deletion of *Sln* did not improve SERCA function nor rescue the CNM phenotype, but did result in exacerbated muscle atrophy and weakness, due to a failure to induce type II fiber compensatory hypertrophy and a reduction in total myofiber count. Mechanistically, our findings suggest that impaired calcineurin activation and resultant decreased expression of stabilin-2, and/or impaired autophagic signaling could be involved. Future studies should examine these possibilities. In conclusion, our study demonstrates the importance of SLN upregulation in combating muscle myopathy in the *Pln*^OE^ mice, and since SLN is upregulated across several myopathies, our findings may reveal SLN as a novel and universal therapeutic target.

## Introduction

The muscle proteins, sarcolipin (SLN) and phospholamban (PLN), are two well-known regulators of sarco(endo)plasmic reticulum Ca^2+^-ATPase (SERCA) pumps [1, 2]. We have recently discovered that type I fiber specific PLN overexpression in mice (*Pln*^OE^) causes severe impairments in SERCA function and a centronuclear myopathy (CNM)-like phenotype in the soleus and gluteus minimus muscles [3]. Along with severe muscle atrophy and weakness, these muscles displayed the three main histological features associated with CNM: 1) increased central nuclei, 2) central aggregation of oxidative activity, and 3) type I fiber predominance and hypotrophy [4]. SLN protein was upregulated 7–9 fold in the soleus and gluteus minimus muscles from *Pln*^OE^ mice compared with wild-type (WT) [3], a feature that is common among other mouse models of muscle disease where SLN is often upregulated [5–12]. However, the physiological role of SLN upregulation in *Pln*^OE^ mice remains unknown.

Co-expression of SLN and PLN at supraphysiological levels in HEK-293 cells results in superinhibition of calcium uptake [13]. Although the exact mechanisms leading to CNM is unclear, aberrant calcium handling [14, 15] has been suggested to have a pathological role. SERCA inhibition and the concomitant rise in [Ca^2+^]_i_ could lead to the generation of reactive oxygen species [16] and activation of the Ca^2+^-dependent calpain proteases [17, 18] ultimately leading to muscle wasting. Since forced over-expression of SLN in skeletal muscle represses SR Ca^2+^ uptake and impairs contractile function [19] and SLN ablation enhances Ca^2+^ transport and muscle relaxation [20], we hypothesized that preventing the upregulation of SLN protein in *Pln*^OE^ mice may mitigate CNM pathology and combat the muscle atrophy and weakness that occurs. To test this hypothesis, we crossed *Sln*-null (*Sln*^KO^) mice [21] with heterozygous *Pln*^OE^ mice [3] and examined the effect on SERCA function, CNM pathology, soleus muscle mass, and contractile function.

## Methods

### Mice

The *Pln*^OE^ and *Sln*^KO^ mice have been described previously [3, 21]. To direct type I fiber specific *Pln* overexpression, the *Pln* transgene was attached to the β-myosin heavy chain (MHC) promoter [22], which directs high levels of type I skeletal-muscle specific transgene expression [23–25]. FVB/N *Pln*^OE^ mice were resuscitated from cryopreserved embryos by the mmRRC (000067-MU) to generate a breeding colony with WT FVB/N mice in our facility. Since the *Sln*^KO^ mice were generated onto a C57BL/6J background [21], we backcrossed heterozygous male FVB/N *Pln*^OE^ animals with either C57BL/6J *Sln*^KO^ or WT littermate females for 6 generations to generate *Pln*^OE^/*Sln*^KO^, *Pln*^OE^, and WT control lines on a C57BL/6J background. All *Pln* overexpressing, with or without *Sln*, were heterozygous for *Pln*. With this breeding strategy, WT and *Pln*^OE^ littermate mice were cousins to *Pln*^OE^/*Sln*^KO^ mice. All animals used in the study were adult mice (WT, 6.2 ± 0.1 months; *Pln*^OE^, 6.1 ± 0.7 months; *Pln*^OE^/*Sln*^KO^, 6.1 ± 0.6 months). Animals were housed in an environmentally controlled room with a standard 12:12-hour light-dark cycle and allowed access to food and water *ad libitum*. All animal procedures were reviewed and approved by the Animal Care Committee of the University of Waterloo and are consistent with the guidelines established by the Canadian Council on Animal Care.

### SERCA activity and Ca^2+^ uptake

Ca^2+^-dependent SERCA activity was assessed in soleus homogenates over Ca^2+^ concentrations ranging from *p*Ca 7.2 to 5.4 in the presence of the Ca^2+^ ionophore A23187 (Sigma C7522) using a spectrophotometric plate reader assay as previously described [26]. Maximal SERCA activity was taken from the raw data and SERCA activity-*p*Ca curves were generated with GraphPad Prism^TM^ (CA, USA) by non-linear regression curve fitting using an equation for a general cooperative model for substrate activation. Ca^2+^ uptake was measured at a free [Ca^2+^] of 1500 nM in soleus homogenates in the presence of precipitating anion, oxalate, using the fluorescent dye Indo-1 and a spectrofluorometer equipped with dual-emission monochromators [27].

### *In vitro* muscle contractility

WT, *Pln*^OE^, and *Pln*^OE^/*Sln*^KO^ mice were sacrificed by cervical dislocation, and the intact soleus muscles were removed and placed into a bath with oxygenated Tyrode solution (95% O_2_, 5% CO_2_) containing 121 mM NaCl_2_, 5 mM KCl, 24 mM NaHCO_3_, 1.8 mM CaCl_2_, 0.4 mM NaH_2_PO_4_, 5.5 mM glucose, 0.1 mM EDTA, and 0.5 mM MgCl_2_, pH 7.3, that was maintained at 25°C. Muscles were situated between two platinum electrodes and force was electrically evoked and assessed across a range of stimulation frequencies from 1 to 100 Hz using a biphasic stimulator (Model 710B, Aurora Scientific, Inc., ON, Canada). Data were analyzed using Dynamic Muscle Control Data Acquisition software (Aurora Scientific). Specifically, peak isometric force amplitude (mN) and the maximal rates of force development (+dF/dt) and relaxation (−dF/dt) were determined during a twitch and across the range of stimulation frequencies. Peak isometric force was then normalized to muscle weight (mN/g).

### Antibodies

Primary antibodies against PLN (2D12), phosphorylated-NFATc1 (PA5-38301), total NFATc1 (MA3-024), SERCA2a (MA3-919), ryanodine receptor 1 (RyR, MA3-925), dihydropyridine receptor α1 subunit (DHPR, MA3-920), and calsequesterin I and II (CSQ, MA3-913) were obtained from Pierce Antibodies. The primary antibody directed against SLN was generated by Lampire Biological Laboratories (PA, USA) [28]. LC3B (2775) and p62 (GP62-C) antibodies were obtained from Cell Signaling Technology (MA, USA) and Progen Biotechnik (Heidelberg, Germany), respectively. The primary antibody against actin (A2066) and stabilin-2 (orb158499) were obtained from Sigma Aldrich (MO, USA), and Biorbyt (CA, USA), respectively. SERCA1a antibody (A52) was a kind gift from Dr. David MacLennan (University of Toronto) [29]. The primary antibodies against MHCI (BA-F8), MHCIIa (SC-71), and MHCIIb (BF-F3) were obtained from Developmental Studies Hybridoma Bank (IA, USA). Secondary antibodies for western blotting, goat anti-mouse IgG (peroxidase conjugated) and goat anti-rabbit IgG (peroxidase conjugated) were obtained from Santa Cruz Biotechnology (TX, USA). Secondary antibodies for immunofluorescence staining, Alexa Fluor 350 anti-mouse IgG_2b_, Alexa Fluor 488 anti-mouse IgG_1_, and Alexa Fluor 555 anti-mouse IgM, were obtained from Molecular Probes (OR, USA).

### Histological, histochemical and immunofluorescent staining

Soleus samples embedded in O.C.T. compound were cut into 10 μm thick serial cross sections with a cryostat (Thermo Electronic, MA, USA) maintained at -20°C. Histological staining included H&E and Van Gieson, and histochemical staining included succinate dehydrogenase (SDH) activity. Images were acquired with a brightfield Nikon microscope linked to a PixeLink digital camera and quantified with ImageJ software (NIH, MA, USA). Immunofluorescence analysis of MHC expression was previously described [30] and performed with primary antibodies against MHCI, MHCIIa, and MHCIIb. Fibers that were not positively stained with MHCI, IIa, or IIb antibodies were considered as type IIX fibers. Slides were visualized with an Axio Observer Z1 fluorescent microscope equipped with standard red, green, blue filters, an AxioCam HRm camera, and AxioVision software (Carl Ziess, Oberkochen, Germany). Quantification of fiber distribution and cross-sectional area (CSA) was performed using imageJ software.

### Western blotting

Western blotting was performed to determine expression levels of SLN, PLN, phosphorylated-PLN, SERCA1a, SERCA2a, RyR, DHPR, CSQ, phosphorylated-NFATc1, total NFATc1, stabilin-2, LC3, and p62. Solubilized proteins from tissue homogenates were separated using tricine based SDS-PAGE (13% total acrylamide for PLN and SLN) or standard SDS-PAGE (7.5% total acrylamide for phosphorylated- NFATc1, total NFATc1, SERCA1a, SERCA2a, RyR, DHPR, and CSQ,; 12% total acrylamide for p62 and LC3). Separated proteins were then transferred onto 0.2 μm PVDF membranes for all analyses except for SLN, which utilized nitrocellulose membranes. Membranes were then immunoprobed with their corresponding primary antibodies and subsequently immunoprobed with horseradish peroxidase-conjugated secondary antibodies. Antigen-antibody complexes were detected by SuperSignal West Femto^TM^ substrate (Pierce, Thermo Fisher Scientific Inc., MA, USA) for SLN, phosphorylated NFAT, and stabilin-2; Luminata Forte^TM^ (Millipore, MA, USA) for PLN, phosphorylated PLN, and RYR; and ECL Western Blot Substrate (BioVision, MA, USA) for total NFAT, SERCA1a, SERCA2a, CSQ, DHPR, LC3 and p62. Quantification of optical densities was performed using GeneTools (Syngene, MD, USA) and values were normalized to the densitometric sum of all bands visualized through ponceau staining or actin. For RyR, DHPR, and CSQ analyses, the same PVDF membrane was used and cut into their corresponding strips (RyR > 250 kDa; DHPR 100–250 kDa; and CSQ < 100 kDa).

### Electron microscopy

To examine the triad structures in the soleus muscles of WT, *Pln*^OE^, and *Pln*^OE^/*Sln*^KO^ mice, we performed transmission electron microscopy (TEM). Briefly, muscle samples (1 cubic mm each) were fixed immediately in 2.5% glutaraldehyde (EM grade) in 0.2M phosphate buffer (pH 7.4), overnight at 4°C. Subsequently, samples were postfixed in 1% osmium tetraoxide in the same buffer, dehydrated in a graded acetone series, and embedded in an epon-aldarite resin. Semithin sections (0.74 μm) were stained with toluidine blue and placed on a hot plate for 30 seconds to determine orientation of samples. Ultrathin longitudinal sections (90 nm) were cut on a Leica Reichart Ultracut E microtome (Leica Microsystem, Wetzlar, Germany), double contrasted with uranyl acetate (50% in methanol) and lead citrate (0.4% w/v in boiled H_2_O), and viewed in a CM 10 Philips TEM.

### Proteolytic enzyme activity assays

Caspase-3 and cathepsin-B/L activity were determined in soleus muscle homogenates using the substrates Ac-DEVD-AMC (Alexis Biochemicals) and z-FR-AFC (Enzo Life Sciences, NY, USA), respectively, whereas calpain and 20S proteasome activity were determined using Suc-LLVY-AMC (Enzo-Life Sciences) [3]. These fluorogenic substrates are weakly fluorescent but yield highly fluorescent products following proteolytic cleavage by their respective proteases. Fluorescence was measured using a SPECTRAmax Gemini XS microplate spectrofluorometer (Molecular Devices, Sunnyvale, CA) with excitation and emission wavelengths of 360 nm and 440 nm (caspase-3), 380 nm and 460 nm (calpain and 20S proteasome), or 400 nm and 505 nm (cathepsin), respectively. To distinguish between calpain and 20S proteasome activity, the specific inhibitors Z-Leu-Leu-CHO (calpain; Enzo-Life Sciences) and epoxomicin (20S proteasome; Cayman Chemical, MI, USA) were incubated and the difference in fluorescence from homogenates incubated with and without the respective inhibitors was measured. All proteolytic activities were normalized to total protein content and expressed as fluorescence intensity in arbitrary units per mg of protein.

### Daily activity and food intake measurements

Daily food consumption (g/day) and cage activity were measured using a comprehensive laboratory animal monitoring system (CLAMS; Oxymax series; Columbus Instruments, Columbus, OH, USA) as previously described [31]. This system is equipped with a feed scale for monitoring mass of food consumed, as well as *X*- and *Z*-axes infrared photocell detectors that allow for monitoring ambulatory activity (when 2 adjacent *x*-axis beams were broken in succession) and total cage activity. Mice were placed in the CLAMS for a 3-day period for 3 separate trials and had free access to food and water.

### Statistics

All values are presented as means ± standard error (SEM). Statistical significance was set to *p* ≤ 0.05. Most comparisons between WT, *Pln*^OE^, and *Pln*^OE^/*Sln*^KO^ mice were performed using a one-way ANOVA with a Tukey’s post-hoc test or a Games-Howell post-hoc test to control for unequal variances. A two-way ANOVA was used to examine the force frequency curves.

## Results

### Effect of *Sln* ablation on SERCA function

**Fig 1:**
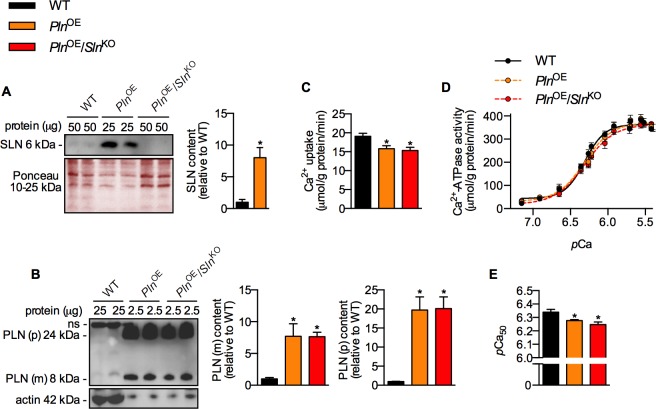
*Sln* deletion does not rescue SERCA function in *Pln*^OE^ mice. (A) Western blot analyses showing successful removal of the 8-fold upregulation in SLN protein (n = 6 per genotype). Ponceau staining ensured equal loading. (B) *Pln*^OE^ and *Pln*^OE^/*Sln*^KO^ soleus muscles displayed similar levels of monomeric (m) and pentameric (p) PLN overexpression compared with WT (n = 6 per genotype). Values were normalized to actin. (C) Rates of Ca^2+^ uptake were similarly reduced in the *Pln*^OE^ and *Pln*^OE^/*Sln*^KO^ soleus muscles compared with WT (n = 6–7, per genotype). (D) Representative SERCA activity-*p*Ca curves measured in soleus homogenates (n = 6–7, per genotype). (E) SERCA’s apparent affinity for Ca^2+^, presented as *p*Ca_50_, was similarly reduced in the *Pln*^OE^ and *Pln*^OE^/*Sln*^KO^ soleus muscles compared with WT (n = 6–7, per genotype). *p*Ca_50_ is the negative logarithm of the Ca^2+^ concentration required to attain half-maximal SERCA activity rate. *Significantly different from WT using a Student’s t-test (A), and a one-way ANOVA with a Tukey’s post-hoc (B, C and E), *p* ≤ 0.05. All values are means ± SEM.

Similar to the FVB/N line [3] we observed an 8-fold upregulation of SLN protein in the *Pln*^OE^ soleus, and we confirmed the lack of SLN protein after genetic deletion of *Sln* (Fig 1A). Removal of *Sln* did not alter the level of monomeric or pentameric PLN overexpression (Fig 1B). Unexpectedly, the rates of Ca^2+^ uptake, and SERCA’s apparent affinity for Ca^2+^ were similarly reduced in the *Pln*^OE^ and *Pln*^OE^/*Sln*^KO^ soleus muscles compared with WT (Fig 1C–1E).

### PLN phosphorylation and expression of other Ca^2+^regulatory proteins

**Fig 2:**
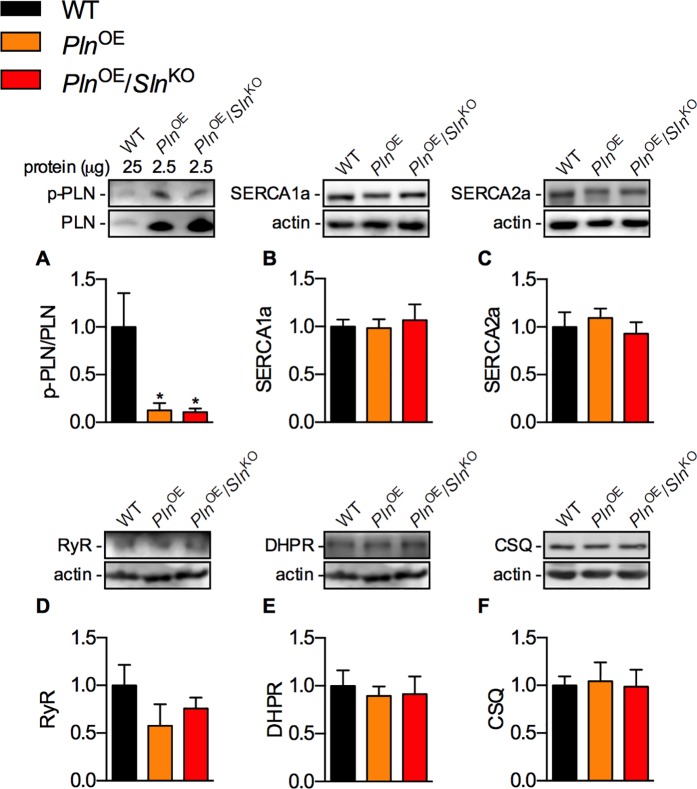
PLN phosphorylation and expression levels of other Ca^2+^ regulatory proteins. (A) Western blot analyses demonstrating that monomeric p-PLN/PLN ratio is significantly lower in both *Pln*^OE^ and *Pln*^OE^/*Sln*^KO^ soleus muscles compared with WT (n = 5–6 per genotype). Western blotting for other major Ca^2+^ regulatory proteins SERCA1a (B, 110 kDa), SERCA2a (C, 110 kDa), RyR (D, 565 kDa), DHPR (E, 170 kDa), and CSQ (F, 63 kDa) did not reveal any significant differences across genotypes (n = 5–8 per genotype, with a 6 μg load for all proteins). *Significantly different from WT using a a one-way ANOVA with a Tukey’s post-hoc, *p* ≤ 0.05. All values are means ± SEM.

As with total PLN expression, only 2.5 μg of protein from soleus homogenates was required to detect phosphorylated PLN (p-PLN) in the *Pln*^OE^ and *Pln*^OE^/*Sln*^KO^ mice compared to the 25 μg of protein required from WT mice (Fig 2A). The p-PLN/PLN ratio was significantly lower in both *Pln*^OE^ and *Pln*^OE^/*Sln*^KO^ mice compared with WT, but there were no differences between *Pln*^OE^ and *Pln*^OE^/*Sln*^KO^ mice (Fig 2A). Assessment of the other major Ca^2+^ regulatory proteins including SERCA1a, SERCA2a, DHPR, RyR, and CSQ did not reveal any significant differences (Fig 2B–2F).

### CNM phenotype in *Pln*^*OE*^ and *Pln*^*OE*^*/Sln*^*KO*^ mice

**Fig 3:**
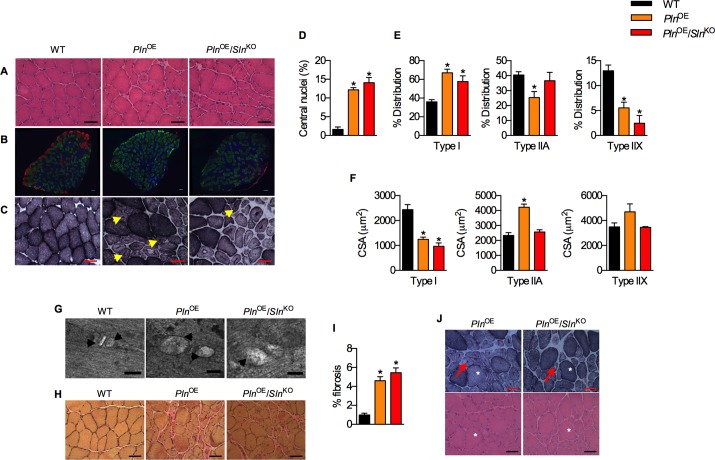
SLN does not contribute to CNM pathology in *Pln*^OE^ mice. Representative H&E (A), immunofluorescent (B), and SDH (C) stained sections showing the CNM phenotype of elevated central nuclei (D), type I fiber predominance (E) and type I fiber hypotrophy and type II fiber hypertrophy (F), and central aggregation of oxidative activity (yellow arrows in C) (n = 4–6 per genotype). (G) Transmission electron micrographs illustrating triad disruptions with swollen sarcoplasmic reticulum in both the *Pln*^OE^ and *Pln*^OE^/*Sln*^KO^ soleus muscles. Arrowheads point to the swollen sarcoplasmic reticulum with corresponding area. Van-Giesons staining (H) quantified with imageJ (I) reveals greater endomysial fibrosis the *Pln*^OE^ and *Pln*^OE^/*Sln*^KO^ soleus muscles compared with WT (n = 4 per genotype). (J) Representative SDH-stained and H&E-stained sections displaying core-like aspects in the *Pln*^OE^ and *Pln*^OE^/*Sln*^KO^ soleus muscles that cannot be explained by the presence of an artifact or vacuole. Arrows point to the core-like aspect and asterisks depict the same fiber within serial sections. *Significantly different from WT using a one-way ANOVA with a Tukey’s post-hoc, *p* ≤ 0.05. All values are means ± SEM. CSA, cross-sectional area. All scale bars are set to 50 μm except for (G) where scale bars are set to 100 nm.

To determine whether genetic deletion of SLN would influence the CNM phenotype, we performed: 1) H&E staining to examine the percentage of central nuclei, 2) SDH staining to look for central aggregation of oxidative activity, and 3) immunofluorescent staining to assess type I fiber distribution and size. Both the *Pln*^OE^ and *Pln*^OE^/*Sln*^KO^ soleus muscles exhibited a CNM-like phenotype with an increase in central nuclei, central aggregation of oxidative activity, and type I fiber predominance and hypotrophy (Fig 3A–3F). Consistent with other models of CNM and biopsies from CNM patients [32–35], TEM analyses revealed triad abnormalities with swollen sarcoplasmic reticulum membranes in both the *Pln*^OE^ and *Pln*^OE^/*Sln*^KO^ soleus muscles (Fig 3G). In addition to this and in agreement with our initial characterization of the *Pln*^OE^ mice [3], endomysial fibrosis and core-like lesions were evident in the *Pln*^OE^ and *Pln*^OE^/*Sln*^KO^ soleus (Fig 3H–3J). Collectively, these data suggest that *Sln* deletion had very little effect on the overall CNM phenotype that occurs with PLN overexpression.

### Effect of *Sln* deletion on muscle size and contractile function

**Fig 4:**
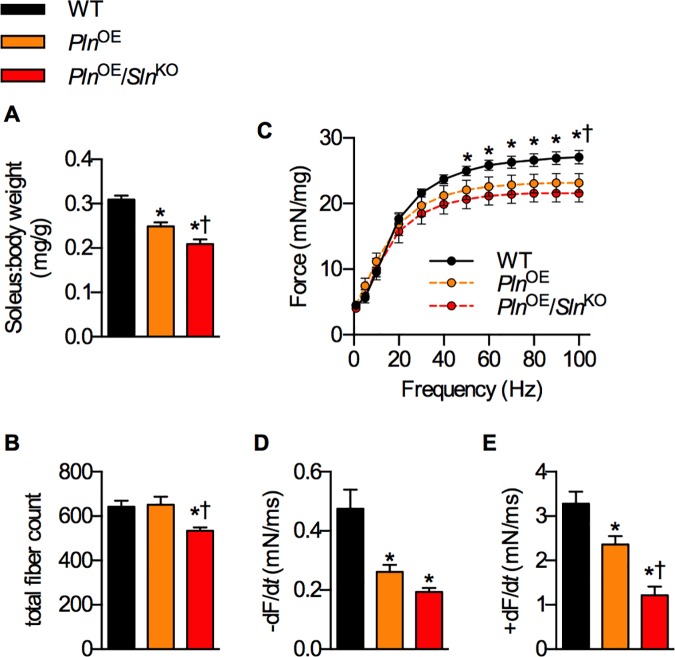
Without SLN upregulation, *Pln*^OE^ soleus muscles are smaller and weaker. (A) soleus:body weight ratios (mg/g) across genotype (n = 18–23 per genotype). (B) Total myofiber count in soleus muscle sections counted after immunofluorescent staining (n = 6 per genotype). (C) Mass-specific force-frequency curve analysis in isolated soleus muscles (n = 6–7 per genotype). Rates of relaxation (-dF/dt, D) and force development (+dF/dt, E) measured for a single twitch (n = 6–7 per genotype). For (A, B, D and E), *significantly different from WT; ^†^significantly different from *Pln*^OE,^ using a one-way ANOVA *p* ≤ 0.05. For C, *significantly different from *Pln*^OE^/*Sln*^KO^; ^†^significantly different from *Pln*^OE,^ using a two-way ANOVA *p* ≤ 0.05. All values are means ± SEM.

The absence of SLN exacerbated the soleus muscle atrophy seen with PLN overexpression [3, 22] as the soleus:body weight ratios were smallest in the *Pln*^OE^/*Sln*^KO^ mice compared with both *Pln*^OE^ and WT (Fig 4A). Furthermore, the type IIA fibers from *Pln*^OE^/*Sln*^KO^ mice failed to undergo the typical compensatory hypertrophy observed in the *Pln*^OE^ mice (Fig 3F and reference [3]), and the type IIX fibers showed a similar pattern (Fig 3F); however the one-way ANOVA did not indicate statistical significance (*p* = 0.11). There was also a significant reduction in total fiber count in the *Pln*^OE^/*Sln*^KO^ soleus muscles compared with both *Pln*^OE^ and WT mice (Fig 4B). Soleus muscles from *Pln*^OE^/*Sln*^KO^ mice displayed significant reductions in mass-specific force from 50-100Hz stimulation compared with WT (Fig 4C). In contrast, the *Pln*^OE^ soleus muscles were only significantly weaker than WT at 100 Hz (Fig 4C). Furthermore, comparisons between *Pln*^OE^ and *Pln*^OE^/*Sln*^KO^ soleus force-frequency curves using a two-way ANOVA reveals a significant main effect of genotype (*p* = 0.02), which suggests that the *Pln*^OE^/*Sln*^KO^ soleus muscles are weaker than the *Pln*^OE^ soleus muscles. With respect to twitch kinetics, the rates of relaxation (Fig 4D) and force development (Fig 4E) were reduced in the *Pln*^OE^ and *Pln*^OE^/*Sln*^KO^ soleus muscles compared with WT, with the rates of force development being significantly slower in *Pln*^OE^/*Sln*^KO^ compared with *Pln*^OE^. Importantly, body weight, daily food intake, and daily activity were not different between groups (Table 1), and therefore cannot explain the worsened soleus muscle atrophy and weakness.

**Table 1:** Body weight, daily activity and daily food intake measures from WT, *Pln*^OE^, and *Pln*^OE^/*Sln*^KO^ mice.

Parameter	WT	*Pln*^OE^	*Pln*^OE^/*Sln*^KO^
Body weight (g)	35.9 ± 0.8	37.5 ± 0.8	36.0 ± 1.1
Food intake (g/d)	4.9 ± 0.5	5.2 ± 0.3	4.7 ± 0.4
Total activity (counts/d)	15,354 ± 1,073	11,070 ± 1,690	12,072 ± 1,468
Ambulatory activity (counts/d)	5,435 ± 680	3,665 ± 681	3,950 ± 641

Values are means ± SEM (n = 18–23, for the average body weight of *Pln*^WT^/*Sln*^WT^, *Pln*^OE^/*Sln*^WT^, and *Pln*^OE^/*Sln*^KO^ mice; n = 6 per genotype for daily food consumption, ambulation, and total activity). Ambulatory activity counts were when the mouse crossed 2 adjacent x-axis infrared beams in succession. One-way ANOVA revealed no significant effect of genotype for body weight (*p* = 0.37), food intake (*p* = 0.48), total activity (*p* = 0.12), or dual beam activity (*p* = 0.17).

### Fiber type distribution

In response to *Pln* overexpression in the slow-twitch type I fibers, the type II fibers decrease in proportion while the type I fibers increase [3, 22]. A similar result was observed in this study with significant reductions in both type IIA and type IIX fiber percentages in *Pln*^OE^ mice compared with WT (Fig 3E). In contrast, the type IIA fiber distribution in the *Pln*^OE^/*Sln*^KO^ was not different from WT (Fig 3E) suggestive of a delayed fast-to-slow fiber type shift.

### NFATc1 phosphorylation and stabilin-2 expression as markers of calcineurin signaling

**Fig 5:**
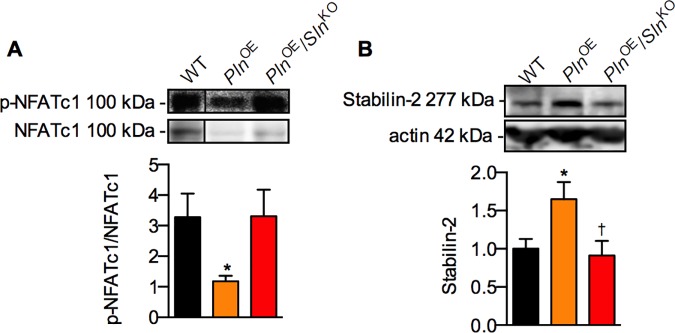
Elevated NFATc1 phosphorylation status (A) and a failure to promote stabilin-2 expression (B) indicates blunted calcineurin signaling in *Pln*^OE^/*Sln*^KO^ mice. * Significantly different from WT using a one-way ANOVA with a Games-Howell post-hoc for unequal variances (A, n = 7–12 per genotype), or a Tukey’s post-hoc test (B, n = 9–10 per genotype); ^†^significantly different from *Pln*^OE^, *p* ≤ 0.05. All values are means ± SEM.

To explain the lack of type II fiber hypertrophy and the delay in the fast-to-slow fiber type shift in the absence of *Sln*, we focused on calcineurin, the Ca^2+^-calmodulin dependent serine/threonine phosphatase known to regulate these cellular adaptations [36–38] and, perhaps more importantly, shown to be responsive to SLN [39]. When examining the phosphorylation status of nuclear factor of activated T-cells cytoplasmic 1 (NFATc1) as a marker of calcineurin signaling, compared with WT we found that NFATc1 phosphorylation relative to total NFATc1 was significantly lower in *Pln*^OE^ but not *Pln*^OE^/*Sln*^KO^ soleus (Fig 5A). Calcineurin-mediated activation of NFATc1 increases the expression of stabilin-2; a phosphatidylserine receptor required for myoblast fusion [40]. Corresponding well with the significant dephosphorylation of NFATc1 only in the *Pln*^OE^ mice, we found a significant elevation in stabilin-2 expression only in *Pln*^OE^ soleus compared with WT (Fig 5B). Stabilin-2 expression was also higher in the *Pln*^OE^ soleus compared to the *Pln*^OE^/*Sln*^KO^ soleus.

### Assessment of the major proteolytic pathways and autophagy

**Fig 6:**
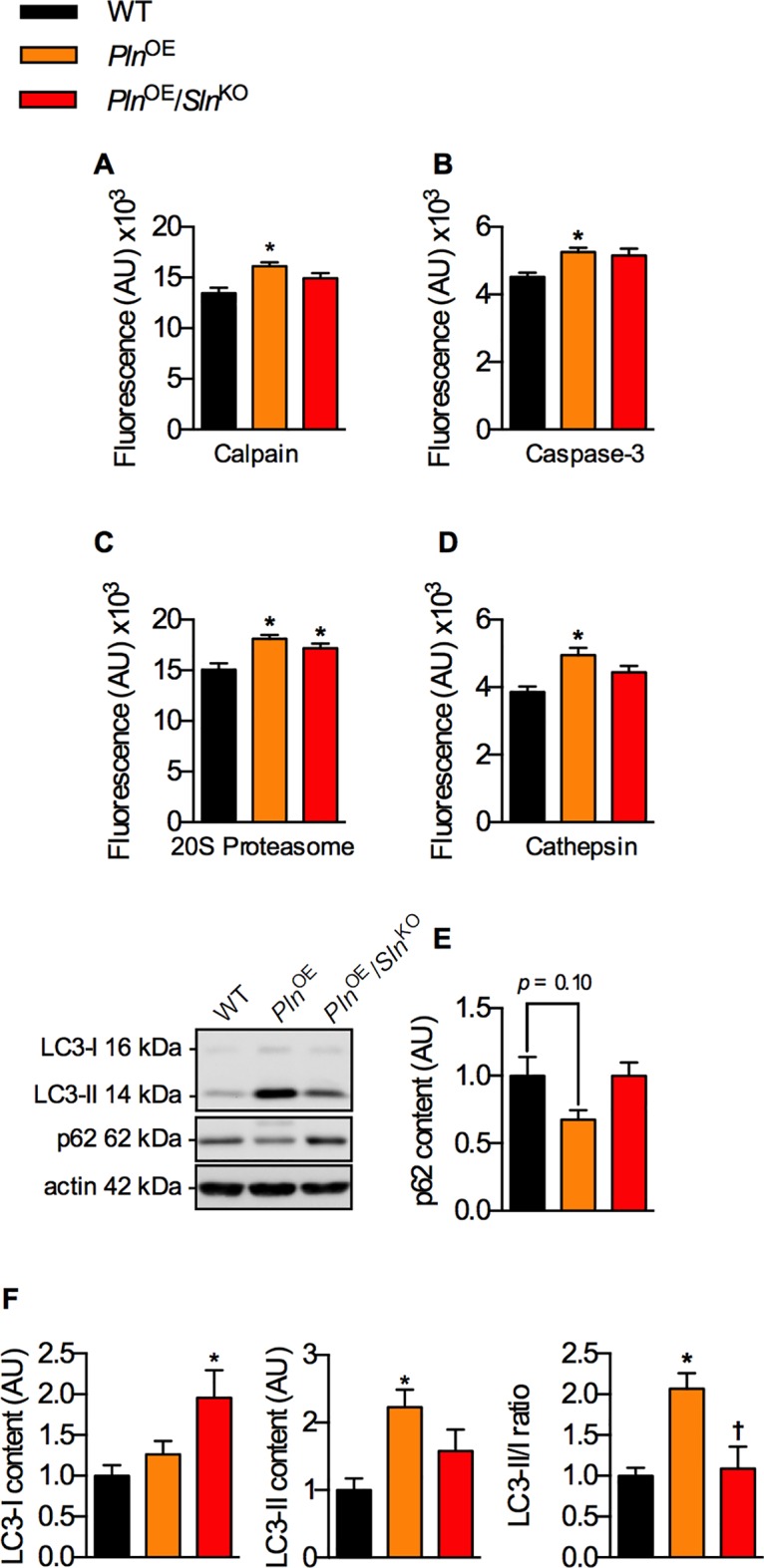
*Sln* deletion does not augment proteolytic activity and autophagic signaling in response to *Pln* overexpression. Calpain (A), caspase-3 (B), and 20S proteasome activity assays (C). Enhanced autophagy revealed by increased cathepsin activity (D), decreased p62 levels (E) and increased LC3-II/I content (F) in *Pln*^OE^ mice is attenuated in *Pln*^OE^/*Sln*^KO^ mice. *Significantly different from WT, ^†^significantly different from *Pln*^OE^ using a one-way ANOVA and a Tukey’s post-hoc test, *p* ≤ 0.05. For proteolytic measures, n = 6–7; for p62, LC3-I and–II content, n = 11–12 and optical densities were normalized to actin. All values are means ± SEM.

Next, we tested whether elevated proteolytic activities could also contribute to the exacerbated soleus muscle atrophy seen in the *Pln*^OE^/*Sln*^KO^ mice. Consistent with our previous study [3], calpain, caspase-3, 20S proteasome and cathepsin activities were all significantly elevated in the *Pln*^OE^ soleus compared with WT; however, none of these were augmented further in the *Pln*^OE^/*Sln*^KO^ soleus (Fig 6A–6D). In fact, only the 20S proteasome was significantly elevated in the *Pln*^OE^/*Sln*^KO^ soleus compared with WT. Moreover, a trending reduction in p62 content (*p* = 0.10) and a significant increase in the LC3-II/I ratio in the *Pln*^OE^ soleus, combined with the elevated cathepsin activity, indicate greater autophagic signaling compared with WT (Fig 6E and 6F); however, we did not observe this in the *Pln*^OE^/*Sln*^KO^ mice. Taken together, these results suggest that proteolytic activity and autophagic signaling were not enhanced in *Pln*^OE^/*Sln*^KO^ soleus and cannot explain the exacerbated muscle atrophy caused by *Sln* ablation.

## Discussion

In this study, we examined the physiological role of SLN upregulation in the *Pln*^OE^ mouse model of CNM by generating the *Pln*^OE^/*Sln*^KO^ mouse line. We hypothesized that SLN ablation would alleviate the CNM pathology, muscle atrophy, and weakness in this model through improvements in SERCA function. However, in contrast to our hypotheses, we did not observe any improvements in SERCA function or CNM phenotype in the *Pln*^OE^/*Sln*^KO^ soleus, and found that *Sln* deletion actually exacerbated the soleus muscle atrophy and weakness by preventing the compensatory type II fiber hypertrophy and reducing the total myofiber count. In addition, rates of force development were slower in the *Pln*^OE^/*Sln*^KO^ soleus compared with those from *Pln*^OE^ mice, which could be due to excitation-contraction (E-C) coupling impairments related to triad structural defects.

In a previous study with *Sln* overexpressing mice, SLN was shown to stimulate calcineurin [39], a Ca^2+^-dependent phosphatase that is implicated in muscle growth [36, 38, 41] and promotion of the slow-oxidative phenotype [36, 41–45]. Pharmacological inhibition of calcineurin prevents myofiber hypertrophy and the fast-to-slow fiber type transition in plantaris muscles that have been mechanically overloaded after synergist ablation of the soleus and gastrocnemius [36, 41, 46]. We propose that the *Pln*^OE^ soleus represents a model of intramuscular overload, whereby the type I fiber hypotrophy results in overload of the type II fibers within the soleus causing them to hypertrophy and transition towards the slow oxidative phenotype [3]. However, in the absence of SLN upregulation, the fast-to-slow fiber type shift and type II fiber hypertrophy are attenuated in the *Pln*^OE^ mice.

A failure to dephosphorylate NFAT and increase expression of stabilin-2 are indicative of impaired calcineurin signaling in the *Pln*^OE^/*Sln*^KO^ mice. It is well accepted that calcineurin activation is important for controlling muscle fibre type in adaptive muscle remodeling so lowered calcineurin activation can at least partly explain the blunted fast-to-slow fiber type shift in the *Pln*^OE^/*Sln*^KO^ soleus. On the other hand, there is some discrepancy regarding calcineurin’s role in stimulating muscle growth. Specifically, some studies have failed to replicate the hypertrophic effect of calcineurin in the overloaded plantaris [47, 48], and genetically modified mice, characterized with either calcineurin inhibition or over-activation, display no effects on myofiber size or muscle mass [45, 49–51]. Nonetheless, calcineurin’s role in myoblast fusion is well established [52–54] and recent evidence has shown that calcineurin activation, specifically through NFATc1, increases the expression of a phosphatidylserine receptor, stabilin-2, and that without stabilin-2, myoblast fusion, myofiber size, and muscle mass are severely reduced [40]. According to the myonuclear domain theory, an increase in the relative amount of nuclei per fibre, via enhanced myoblast fusion to pre-existing myofibers, will increase myofiber size and overall muscle mass [54, 55]. Collectively, our results suggest that the blunted calcineurin signalling in the *Pln*^OE^/*Sln*^KO^ mice reduces stabilin-2 expression and likely impairs myoblast fusion, thereby contributing to the reductions in soleus size, type II fiber CSA, and force generation.

Impairments in stabilin-2 and myoblast fusion could also contribute to the reduction in total fiber count we observed in the *Pln*^OE^/*Sln*^KO^ mice, possibly via impaired muscle regeneration. While calcineurin’s role in muscle regeneration has been well established [56–58], it is important to note that CNMs, including the *Pln*^OE^ mouse, generally do not display signs of extensive muscle regeneration [3, 59]. Alternatively, although excessive autophagy could be detrimental and may lead to muscle atrophy [60], impaired autophagy also results in extensive myofiber degeneration [61]. Therefore, the impaired activation of autophagic signaling in the *Pln*^OE^/*Sln*^KO^ soleus could also contribute to the reductions in total fiber count. Collectively, these results indicate that failure to promote both calcineurin and autophagic signaling in the *Pln*^OE^/*Sln*^KO^ soleus, unlike the *Pln*^OE^ soleus, likely contribute to the hypoplasia and the exacerbated muscle atrophy and weakness in this model.

Upregulated SLN is a hallmark of several muscle diseases [5–12] but it’s unknown whether that response is adaptive or pathogenic. This is the first study to examine the role of SLN upregulation in any form of muscle myopathy and our results indicate that SLN upregulation is an adaptive response that may be required for optimal activation of calcineurin to combat the muscle atrophy and weakness that occurs in the *Pln*^OE^ soleus. In the dystrophic *mdx* mouse model, the affected skeletal muscles show large upregulation of SLN [7], and given the data presented here, this increase in SLN could potentially aid in activating calcineurin and autophagic signaling, both of which have been shown to be promising therapeutic strategies [57, 62–66]. In addition, corticosteroid [63] and high-fat feeding [67] can ameliorate dystrophic pathology in *mdx* mice, and both treatments lead to an increase in SLN expression [9, 31, 68]. Future studies aimed at examining the role of SLN across these different myopathies could further reveal the importance of SLN in overall muscle health.

An unresolved issue is why *Sln* deletion did not result in increased Ca^2+^ uptake and SERCA activity in soleus homogenates, given that *Sln* deletion alone, without *Pln* overexpression, increased the apparent affinity of SERCA for Ca^2+^ by 0.1 *p*Ca units in soleus homogenates compared with WT [20], an effect that we reproduced here (data not shown). Further analysis of PLN’s phosphorylation status or the expression of other major Ca^2+^ regulatory proteins such as SERCA1a, SERCA2a, RyR, DHPR, and CSQ do not provide a likely explanation. *Sln* deletion in *Pln*^OE^ mice primarily affected the type II fibers (ie. attenuated type II fiber hypertrophy and fast-to-slow fiber type shift). Therefore, it is possible that SLN upregulation in soleus from *Pln*^OE^ mice is occurring primarily in the overloaded type II fibers that do not overexpress PLN. In support of this view, previous findings from our laboratory show that SLN is predominantly expressed in type II fibers from human vastus lateralis [28]. If SLN upregulation occurs primarily in the type II fibers, there would be less potential for superinhibition to occur, and any improvement in SERCA function expected in the type II fibers after *Sln* deletion could be masked by the level of PLN overexpression that occurs in the type I fibers, which comprise more than 55% of the muscle fibers. Thus, our study is limited in measuring SERCA function in whole homogenates, but if it was possible to measure SERCA activity specifically in type II fibers then we would expect increased activity in *Pln*^OE^/*Sln*^KO^ compared with *Pln*^OE^, which would correspond with the blunted calcineurin activity we observed in *Pln*^OE^/*Sln*^KO^ soleus.

Our work also sheds light on the differences between PLN and SLN function in skeletal muscle. Our findings showing that PLN overexpression causes myopathy and muscle atrophy whereas SLN upregulation counters it, adds to the notion that PLN and SLN may not be functionally redundant [69]. Indeed, unlike the muscle weakness observed in *Pln*^OE^ mice [3, 22], *Sln*^OE^ mice present with enhanced contractility [70]. It also appears that PLN is different from SLN in its ability to regulate calcineurin signaling since PLN overexpression alone without SLN upregulation resulted in greatly reduced calcineurin signaling. Given that both PLN and SLN are capable of inhibiting SERCA [13, 71, 72], it is surprising that only SLN seems to be a significant regulator of calcineurin. Similarly, it appears that SLN but not PLN is capable of altering autophagy in skeletal muscle since PLN overexpression alone without SLN upregulation did not increase autophagic signaling. We speculate that SLN’s influence on energy expenditure [73] may have a role in activating autophagy, whereas PLN may not have the same influence on ATP consumption in muscle [74], but this needs to be explored further.

In summary, we have found that SLN is upregulated in the soleus muscles from PLN overexpressing mice to combat muscle atrophy and weakness by promoting the compensatory type II fiber hypertrophy and maintaining total myofiber number through calcineurin activation and autophagic signaling. We did not find any alterations in the CNM phenotype in the *Pln*^OE^/*Sln*^KO^ soleus, which suggests that SLN likely has no contribution to CNM pathology. Increased SLN expression is a hallmark of muscle disease, which may represent an adaptive response that is necessary for muscle growth and remodeling and future studies should determine whether SLN provides a novel therapeutic target for other neuromuscular disorders.

## Supporting information

S1 Dataset Minimal dataset for the study. (XLSX)
